# Astroglia as a cellular target for neuroprotection and treatment of neuro‐psychiatric disorders

**DOI:** 10.1002/glia.23136

**Published:** 2017-03-16

**Authors:** Beihui Liu, Anja G. Teschemacher, Sergey Kasparov

**Affiliations:** ^1^ School of Physiology, Pharmacology and Neuroscience University of Bristol, University Walk BS8 1TD United Kingdom; ^2^ Institute for Chemistry and Biology Baltic Federal University Kaliningrad Russian Federation

**Keywords:** astrocytes, astrocytic dysfunction, neurodegenerative disease, therapeutic targets

## Abstract

Astrocytes are key homeostatic cells of the central nervous system. They cooperate with neurons at several levels, including ion and water homeostasis, chemical signal transmission, blood flow regulation, immune and oxidative stress defense, supply of metabolites and neurogenesis. Astroglia is also important for viability and maturation of stem‐cell derived neurons. Neurons critically depend on intrinsic protective and supportive properties of astrocytes. Conversely, all forms of pathogenic stimuli which disturb astrocytic functions compromise neuronal functionality and viability. Support of neuroprotective functions of astrocytes is thus an important strategy for enhancing neuronal survival and improving outcomes in disease states. In this review, we first briefly examine how astrocytic dysfunction contributes to major neurological disorders, which are traditionally associated with malfunctioning of processes residing in neurons. Possible molecular entities within astrocytes that could underpin the cause, initiation and/or progression of various disorders are outlined. In the second section, we explore opportunities enhancing neuroprotective function of astroglia. We consider targeting astrocyte‐specific molecular pathways which are involved in neuroprotection or could be expected to have a therapeutic value. Examples of those are oxidative stress defense mechanisms, glutamate uptake, purinergic signaling, water and ion homeostasis, connexin gap junctions, neurotrophic factors and the Nrf2‐ARE pathway. We propose that enhancing the neuroprotective capacity of astrocytes is a viable strategy for improving brain resilience and developing new therapeutic approaches.

## 1 INTRODUCTION

The central nervous system (CNS) represents a very challenging target for therapeutic interventions. Even though numerus centrally acting drugs are currently in use, these are largely molecules discovered decades ago, sometimes with only minor modifications. It is generally accepted that, for many diseases, effective therapies are lacking and that many of the currently used drugs are only used due to the lack of better ones, in spite of their adverse effects. For decades, the logic for pursuing a potential drug target in the brain was its association with processes localized to neurons, sometimes more and sometimes less specifically aimed at a particular neuronal population. To some extent that reflected the general “neurocentrism” in neuroscience, whereby other components of the brain such as glial and vascular cells were seen as irrelevant. More recently, we have learned of a wide range of mechanisms which astrocytes employ to sustain neuronal networks and sometimes directly affect their operation. One could argue that even though targeting processes which are primarily compartmentalized to astrocytes may not lead to a quick modification of the activity of such networks, in the long term, this approach can be better suited for the chronic human diseases. In this review, we first briefly present evidence that dysregulation of astrocytic functions is a common feature of many CNS diseases and then highlight some of the potentially targetable processes in astrocytes which might be of value for future drug development. For recent reviews on the potential drug targets in microglia see (Moller and Boddeke, 2016; Noda, 2014).

## 2 ASTROCYTES IN THE DISEASED BRAIN ARE CENTRAL TO NEUROPATHOLOGY

Considering the pivotal role of astrocytes in brain homeostasis and the strong metabolic cooperation between neurons and astrocytes, one can postulate that astrocytic dysfunction may lead to neurological disease. These diseases share common pathogenic processes, such as oxidative stress, excitotoxicity, metabolic failure or inflammation, many of which are counteracted by astrocytes in the healthy brain. Thus, disease progression is associated with escalating harmful stimuli that eventually exhaust the neuroprotective mechanisms of astrocytes. Even worse, sometimes deleterious pathways may be switched on in astrocytes, directly contributing to the pathology. Some excellent reviews were published on this topic in recent years (Parpura et al., 2012; Pekny et al., 2016; Sofroniew and Vinters, 2010; Verkhratsky and Parpura, 2016).

### 2.1 Alexander disease—a case of “primary” astrocytic disease

A classic example of a “primary” astrocytic disease is Alexander disease (AxD), a human neurological disorder unequivocally caused by a dysfunction of astrocytes due to mutations in the gene encoding glial fibrillary acidic protein (GFAP) (Brenner, Goldman, Quinlan, & Messing, 2009; Messing, Brenner, Feany, Nedergaard, & Goldman, 2012). A characteristic feature of this fatal disorder is the widespread presence of intracellular protein aggregates in astrocytes, called Rosenthal fibers (RF)—bundles of intermediate filaments surrounding irregular deposits of dense material (Herndon, Rubinstein, Freeman, & Mathieson, 1970). RF are composed of mutant GFAP in association with other constituents, especially the small stress proteins B‐crystallin and heat shock protein 27 (Iwaki, Kume‐Iwaki, Liem, & Goldman, 1989). AxD is considered a gain‐of‐function disorder in the sense that the GFAP mutations produce consequences that differ dramatically from those caused by the absence of GFAP (Brenner et al., 2009; Messing et al., 2012). This makes gene therapy based on expression of wild type GFAP in AxD patients impossible since it may instead exacerbate disease by increasing the GFAP load. One of the most notable functional changes in Alexander astrocytes is the decreased glutamate transport across the cell membrane. More than 75% reduction of glutamate transporter 1 (GLT1, also known as excitatory amino acid transporter 2, EAAT2, or solute carrier family 1 member 2, SLC1A2) immunoreactivity was observed in mouse models of AxD and astrocytes in hippocampal CA1 region of human patients show variable to complete loss of immunostaining for EAAT2 (Tian et al., 2010b). EAAT2 is preferentially localized in astrocytes and is the major mediator of glutamate clearance in humans. Reduced glutamate uptake puts neurons at risk of glutamate overload and excitotoxicity, explaining why seizures are common in Alexander disease (Messing et al., 2012).

### 2.2 Other pathologies involving astrocytes

Astrocytic dysfunction has been extensively implicated in the pathogenesis of numerous diseases for which the primary cause has not yet been identified. These include Alzheimer's disease (AD), Amyotrophic lateral sclerosis (ALS), Epilepsy, Huntington's disease (HD), Ischemia/stroke and Parkinson's disease (PD), some of which are listed in (Table 1).

**Table 1:** Evidence for astrocytic dysfunction in neuro‐psychiatric diseases

CNS disorder	Evidence for the dysfunction of astrocytes	Examples
AD	• Intracellular accumulation of Aß in astrocytes • Astroglial degeneration and atrophy • Release of glia‐derived inflammatory molecules • Reactive astrogliosis • Disturbed calcium homeostasis • Upregulated gap junction • Downregulation of EAAT2 which affects glutamate homeostasis and induces excitotoxicity	(Douen et al. 2000; Filous and Silver 2016; Jack et al. 2010; Kuchibhotla et al. 2009; Meda et al. 2001; Parpura et al. 2012)
ALS	• Decreased expression of EAAT2 • Expression of mutant SOD1 • Astroglial degeneration and atrophy • Reactive astrogliosis	(Rossi and Volterra 2009; Turner and Talbot 2008; Valori et al. 2014)
Epilepsy	• Reactive astrogliosis • Upregulation of glutamate dehydrogenase and downregulation of glutamine synthetase • Alterations of K^+^ buffering, calcium signaling and glutamate and water homeostasis • Deficiency in GABAergic inhibition	(Amiry‐Moghaddam et al. 2003; Bedner and Steinhauser 2013; Coulter and Steinhauser 2015; Robel et al. 2015; Robel and Sontheimer 2016)
HD	• Selective expression of mutant huntingtin • Decreased expression of EAAT2 • Downregulation of Kir4.1 channel • Reactive astrogliosis	(Hsiao et al. 2013; Mangiarini et al. 1996; Maragakis and Rothstein 2001)
Ischemia/stroke	• Compromised glutamate, ion and water homeostasis • Reactive astrogliosis	(Liu and Chopp 2015; Zhao and Rempe 2010)
PD	• Selective expression of mutant α‐synuclein, which induces widespread glial activation and neurodegeneration • Excessive production of cytokines and neurotoxic free‐radicals • Reactive astrogliosis	(Adams et al. 2001; Cabezas et al. 2014; Spillantini et al. 1997; Stefanis 2012; Wang et al. 2015)

#### 2.2.1 AD

AD is one of the most common neurodegenerative disorder characterized by progressive memory loss and a range of cognitive deficits (McKhann et al., 1984). Aggregation and deposition of β‐amyloid (Aβ) and the formation of neurofibrillary tangles are classical hallmarks of AD (Hardy and Selkoe, 2002). Aβ deposition in the brain seems to precede neurofibrillary tangle formation, neuronal cell death and subsequent functional decline (Jack et al., 2010). Astrocytes play an important neuroprotective role in AD by internalizing and degrading Aβ peptides, thus helping to avoid formation of the deposits of toxic extracellular Aß (Koistinaho et al., 2004; Kurt, Davies, & Kidd, 1999). The precise mechanism by which astrocytes recognize and degrade Aβ is not known, but apolipoprotein E (APOE), which is almost exclusively expressed in astrocytes, has been proposed to be responsible for this function (Koistinaho et al., 2004). The APOE gene found in humans on chromosome 19 has three loci: APOE‐ε2, APOE‐ε3 and APOE‐ε4. In 1993 it was demonstrated that homozygocity for APOE‐ε4 greatly increases the risk for late onset AD, being almost sufficient to cause it in patients by the age of 80 (Corder et al., 1993). Shortly afterwards it was reported that the other allele, APOE‐ε2, in contrast, is rather “protective” against AD (Corder et al., 1994). These two isoforms of APOE have an opposite effect on the phagocytic activity of astrocytes whereby APOE‐ε2 increases their ability to “digest” synapses while APOE‐ε4 reduces it, making synapses more vulnerable to complement‐mediated degeneration (Klionsky et al., 2016). Literature on the role of APOE in AD is extensive and its detailed revision is outside of the scope of this review.

Current medicines are ineffective and only temporarily alleviate symptoms, or slightly slow down AD progression in some people. Two types of medication are currently approved by the FDA for use against memory loss in AD, acetylcholinesterase inhibitors and memantine. Memantine is classified as an NMDA receptor antagonist, originally developed as anti‐diabetic drug. It is interesting that NMDA receptors on astrocytes and neurons have different subunit compositions and memantine blocks astroglial NMDA receptors with five times lower IC_50_ than those on neurons (Palygin, Lalo, & Pankratov, 2011). Even though therapeutic activity of memantine in AD is subtle, it is still one of the very few drugs clinically approved for moderate‐to‐severe AD.

Recent studies from M. Nedergaard's laboratory opened a very interesting line of thought in this field. It was shown that the extracellular space which is to a large extent, regulated by the subtle changes in the volume of astrocytes has a dramatic effect on the movement of macro‐molecules and their drainage through the so‐called “glymphatic” system (Iliff et al., 2012; Thrane, Rangroo Thrane, Plog, & Nedergaard, 2015). During wakefulness this extracellular trafficking pathway for tracer molecules and Aβ shrinks, but during sleep it opens up, facilitating brain clearance of potentially toxic products. Coordinated expansion of the glymphatic clearance pathway seems to be controlled via norepinephrine receptors on astrocytes (Xie et al., 2013). Therefore, AD could be to some extent seen as a result of failure of the “brain drain” pathway.

Reactive astrogliosis is another well‐known feature of AD (Meda, Baron, & Scarlato, 2001). Astrogliosis tends to be focal in AD such that reactive astrocytes are associated with amyloid plaques and surround them with layers of processes as if forming miniature scars in an attempt to create neuroprotective barriers (Olabarria, Noristani, Verkhratsky, & Rodriguez, 2010). The intensity of astrogliosis increases with progression of AD, while the levels of astrocyte glutamate transporters decline, exposing neurons to additional excitotoxic damage (Simpson et al., 2010). The glutamate transporter EAAT2 is downregulated in AD (Tian, Kong, Lai, Ray‐Chaudhury, & Lin, 2010a). Calcium homeostasis is also affected. Both resting calcium and intracellular calcium waves in astrocytes near plaques are increased, indicating that the astrocyte network contributes to AD pathology (Kuchibhotla, Lattarulo, Hyman, & Bacskai, 2009). Additionally, gap junctions between astrocytes are altered in AD (Nagy, Li, Hertzberg, & Marotta, 1996). Increased glutamate and ATP release has been linked to altered gap junction expression, suggesting that blocking hemichannels in neurons could be neuroprotective in AD (Orellana et al., 2011).

AD is also accompanied by signs of inflammation (Douen et al., 2000). Increased cerebral levels of Aβ peptides and their subsequent deposition lead to the activation of the surrounding microglia and astrocytes (Li et al., 2011). Upon activation, both microglia and astrocytes release pro‐ and anti‐inflammatory mediators, thereby establishing a chronic parenchymal inflammation (Orre et al., 2014). Chronic inflammatory stimulation of astrocytes reduces their capacity to release neurotrophic factors, for example glia‐derived neurotrophic factor, possibly contributing to cognitive decline in AD (Parpura et al., 2012). At later stages, inflammation becomes directly damaging to the brain and glial cytokines and chemokines lead to destruction of axons, dendrites and synapses (Pekny et al., 2016).

Accumulation of Aβ increases oxidative stress (Radi, Formichi, Battisti, & Federico, 2014), possibly due to a decrease in antioxidants and antioxidant enzymes in astrocytes (Canevari, Abramov, & Duchen, 2004; Zhao and Zhao 2013), or mitochondrial dysfunction which occur already at the early stages of AD (Gandhi and Abramov 2012; Kim, Kim, Rhie, & Yoon, 2015).

To sum up, astrocytes may be involved in the pathogenesis of AD at multiple levels. They might be driving neurodegeneration, but also be elements of defense. Multiple neuroprotective pathways residing in astrocytes have not been fully explored in AD.

#### 2.2.2 HD

HD is a genetic neurodegenerative disorder characterized by progressive motor, cognitive and psychiatric decline (Ghosh and Tabrizi, 2015). HD is caused by an expanded chain (more than 36) of glutamines in the N‐terminal region of the huntingtin protein, causing intracellular accumulation and aggregation of mutant huntingtin (mHTT) (Mangiarini et al., 1996). At the cellular level, neurodegeneration in HD is most evident in striatal medium spiny neurons (MSN) (Vonsattel et al., 1985). However, the expression of mHTT in neurons alone cannot recapitulate the key features of HD (Gu et al., 2005). Indeed, mHTT is accumulated in astrocytes, whose function is altered in HD (Shin et al., 2005). Astrocytic glutamate uptake is defective in the R6/2 HD mouse model, where levels of EAAT2 are reduced, leading to increase in striatal extracellular glutamate and excitotoxicity (Maragakis and Rothstein, 2001). Recently, astrocytic Kir4.1 was reported to be significantly downregulated in HD mouse models, independently of overt astrogliosis (Ben Haim et al., 2015). Decreased expression of Kir4.1 K^+^ channels leads to elevated striatal extracellular K^+^ *in vivo* which can result in depolarization of neurons. Genetic restoration of Kir4.1 levels in striatal astrocytes returned extracellular K^+^ and MSN excitability to normal, along with improvement of some motor functions in R6/2 mice (Tong et al., 2014). Recent work confirmed that the loss of astrocytic Kir4.1‐ and EAAT2‐mediated homeostatic functions in R6/2 mice compromises glutamate handling and Ca^2+^ signaling, contributing to MSNs pathology in the striatum (Jiang, Diaz‐Castro, Looger, & Khakh, 2016). It follows, that the loss of astrocytic control over glutamate and potassium extracellular levels may contribute to pathology seen in HD and the proteins affected by HD in astrocytes, such as EAAT2 and Kir4.1 channels, might represent therapeutic targets in HD. The difficulty, however, is that in both cases we would need positive modulators which is usually a more difficult task than development of blockers.

Other astrocytic functions which have been implicated in pathogenesis of HD are release of GABA, trophic factors, and inflammatory signaling (Filous and Silver, 2016). Astrocytes in HD models release less GABA, resulting in impaired tonic extra‐synaptic inhibition (Wojtowicz, Dvorzhak, Semtner, & Grantyn, 2013). Both human and mouse data consistently show increased activation of the NFkB signaling in astrocytes, leading to enhanced inflammation (Hsiao, Chen, Chen, Tu, & Chern, 2013). Inhibition of astrocyte‐mediated TNFα signaling enhanced motor function and reduced aggregates of mutant huntingtin in a mouse model of HD, suggesting that targeting of this pathway may be a viable strategy to slow the progression of HD (Hsiao et al., 2013). Additionally, accumulation of mHTT aggregates in astrocytes reduces secretion of brain derived neurotrophic factor (Wang et al., 2012). These events induce a reactive state in astrocytes, leading to release of the precursor form of NGF which may promote apoptosis of motor neurons (Domeniconi, Hempstead, & Chao, 2007).

Thus, poor astrocytic clearance of glutamate, improper control of extracellular K^+^, and reduced release of neurotrophic factors are plausible contributors to the pathogenesis of HD.

#### 2.2.3 ALS

ALS is an adult‐onset disorder caused by selective degeneration of cortical and spinal motor neurons, leading to progressive paralysis and muscle atrophy (Gordon, 2013). Both familial and sporadic forms of ALS exist, with ∼20% of familial forms associated with dominant mutations in the gene encoding Cu/Zn‐superoxide dismutase (SOD1). The mutated human hSOD1 has been used for generating experimental models of ALS (Turner and Talbot, 2008). Analysis of various types of these models revealed the primary role of astroglia in pathology. Astroglial degeneration and atrophy associated with the loss of function precede neuronal death and occur before the emergence of clinical symptoms (Valori, Brambilla, Martorana, & Rossi, 2014; Verkhratsky, Parpura, Pekna, Pekny & Sofroniew, 2014). When SOD1 was specifically expressed in astrocytes, it made them highly vulnerable to extracellular glutamate and resulted in secretion of several neurotoxic factors. Silencing of mutant hSOD1 in astrocytes markedly decelerated the progression of experimental ALS (Yamanaka et al., 2008).

Another critical pathogenic factor in ALS is the deficient glutamate clearance by astroglia. Selective loss or dysfunction of astrocytic glutamate transporters in spinal cord and cerebral cortical areas might account for the glutamate excitotoxicity to neurons. Genetic deletion of astrocytic EAAT2 in mice caused death of motor neurons, thus replicating some features of ALS (Staats and Van Den Bosch, 2009). In line with this, immunohistochemistry revealed a selective loss of astroglial EAAT2 in the motor cortex and ventral horn of the spinal cord of tissues from patients with sporadic ALS (Rossi and Volterra, 2009). It has been proposed that the reduced activity of glutamate transporters in familial ALS could be a result of the malfunction of SOD1, leading to long‐lasting oxidation of transporter proteins' sulfhydryl groups (Seifert, Schilling, & Steinhauser, 2006; Trotti, Rolfs, Danbolt, Brown, & Hediger, 1999). At the later stages of ALS, reactive astrogliosis as well as the activation of microglial cells become particularly prominent (Turner et al., 2004; Valori et al., 2014).

To summarize, at the initial stages of ALS, compromised astroglial glutamate clearance may be the cause of glutamate excitotoxicity. Later, reactive responses of astrocytes and microglia progress in parallel with the loss of motor neurons (Zhu et al., 2015).

#### 2.2.4 PD

PD, the second most common age‐associated neurodegenerative disorder, affects ∼1% of the population over 60 years of age. Its main histopathological features are the loss of dopaminergic neurons and the presence of α‐synuclein‐containing aggregates (so‐called Lewy bodies) in the substantia nigra (SN) (Spillantini et al., 1997). In addition to the commonly known motor symptoms, PD is accompanied by autonomic dysfunction, cognitive, psychiatric, sensory symptoms and sleep disturbances.

Oxidative stress and mitochondrial dysfunction are probably the key events which cause degeneration and death of dopaminergic neurons in the SN (Adams, Chang, & Klaidman, 2001; Sayre, Smith, & Perry, 2001). Oxidative stress in PD manifests as low levels of the antioxidant glutathione (GSH) (Bharath, Hsu, Kaur, Rajagopalan, & Andersen, 2002), increased lipid peroxidation (Dexter et al., 1989), nucleic acid oxidation (Alam et al., 1997) and increased iron content in the dopaminergic zones of the brain (Sofic, Paulus, Jellinger, Riederer, & Youdim, 1991). Astrocytes are important for the antioxidant protection via secretion of various antioxidant molecules (Sidoryk‐Wegrzynowicz, Wegrzynowicz, Lee, Bowman, & Aschner, 2011). However, in PD, astrocytic protection of neurons is limited, possibly due to a decline in GSH trafficking caused by chronic iNOS induction (Heales, Lam, Duncan, & Land, 2004). Depletion of GSH may facilitate production of reactive oxygen and reactive nitrogen species, causing alterations in neuronal proteins such as α‐synuclein. Furthermore, the nitration of α‐synuclein by reactive nitrogen species significantly enhances the formation of synuclein fibrils *in vitro*, resembling the situation in PD brains (Chinta and Andersen, 2008; Paxinou et al., 2001).

Chronic neuroinflammation is another hallmark of PD pathophysiology. Post‐mortem analyses of human PD patients and experimental animal studies demonstrate activation of glial cells and increases in pro‐inflammatory factors (Wang, Liu, & Zhou, 2015). Although microglia is the major cell type involved in the inflammatory responses, astrocytes are also involved. A suggested scenario is that α‐synuclein aggregation activates microglia, which then leads to activation of astrocytes by pro‐inflammatory cytokines (Saijo et al., 2009). Uncontrolled neuroinflammation caused by the synergic activation of microglia and astrocytes ultimately results in production of neurotoxic factors which trigger death of dopaminergic neurons in the SN (Glass, Saijo, Winner, Marchetto, & Gage, 2010).

#### 2.2.5 Epilepsy

Epilepsy affects more than 50 million people worldwide (Hesdorffer et al., 2011). The main clinical manifestation are seizures, sudden, and unpredictable episodes of abnormal electrical brain activity which can lead to convulsions. Seizures are signs of excessive synchronisation of neuronal activity and the search for anti‐epileptic drugs have been largely concentrated on compounds that affect neurons, for example ion channel blockers or agonists of GABA_A_ receptors. The efficacy of these drugs, old and newly created, has not improved substantially over the past decades and the drugs merely suppress symptoms without treating the underlying processes. Resistance to treatment is also common. There is, therefore, an urgent need for more efficacious medications. Astrocytes might offer some interesting targets here. Specimens from patients with pharmacoresistant temporal lobe epilepsy and animal epilepsy models revealed alterations in expression, localization and function of astrocytic connexins, K^+^ and water channels. In addition, disturbed gliotransmission as well as malfunction of glutamate transporters and of the astrocytic glutamate‐ and adenosine‐converting enzymes—glutamine synthetase and adenosine kinase, respectively—have been documented in epileptic tissues (Coulter and Steinhauser, 2015).

Downregulation of inward‐rectifying Kir4.1 channels in astrocytes in hippocampus of epileptic patients points to impaired K^+^ clearance from the extracellular space and increased seizure susceptibility [reviewed by (Bedner and Steinhauser, 2013)]. Global knockout of Kir4.1 leads to postnatal lethality (Neusch, Rozengurt, Jacobs, Lester, & Kofuji, 2001), whereas conditional Kir4.1 knockout in astrocytes alone is able to trigger epilepsy (Chever, Djukic, McCarthy, & Amzica, 2010; Haj‐Yasein et al., 2011a). In the same vein, mutations or single nucleotide polymorphisms in the genes encoding Kir4.1 are associated with human epilepsy (Bedner and Steinhauser. 2013). Much of Kir4.1 protein co‐localizes with the water channel AQP4 in the astroglial endfeet (Nielsen et al., 1997), suggesting that K^+^ clearance might depend on concomitant transmembrane flux of water. In line with this idea, reduction in perivascular AQP4 was associated with compromised clearance of extracellular K^+^ and impaired K^+^ buffering (Amiry‐Moghaddam et al., 2003). Prolonged seizures occur in AQP4 knockout mice (Binder et al., 2006).

It is unsurprising that excess of extracellular glutamate characteristic of human epileptic tissue can be linked to recurrent seizures and neuronal death (Glass and Dragunow, 1995). In mice, knockout of EAAT2 results in spontaneous seizures and hippocampal pathology. Pharmacological inhibition of EAAT2 reduced the threshold for evoking epileptiform activity (Campbell and Hablitz, 2004; Demarque et al., 2004). Reduced expression of EAAT2 and glutamate‐aspartate transporters (GLAST, SLC1A3) also occurs in a tuberous sclerosis epilepsy model (Wong et al., 2003). However, the studies investigating the functional expression of astrocytic glutamate transporters in human epilepsy are inconsistent. Some studies reported a downregulation of EAAT1 and EAAT2 (Proper et al., 2002), but others reported no significant changes (Eid et al., 2004). For effective removal of excess extracellular glutamate, the transmitter must be sequestered and metabolized once taken up by astrocytes. Glutamate can be de‐hydrogenated into α‐ketoglutarate by glutamate dehydrogenase. Alternatively, glutamate can be converted into glutamine by glutamine synthase and then returned to neurons. Loss of this astrocyte‐specific enzyme is found in epilepsy (Seifert and Steinhauser, 2013). A likely consequence is that the shortage of glutamine can affect the pool of GABA which is synthesized from glutamate in the inhibitory neurons, thus weakening inhibition and precipitating seizures (Alvestad et al., 2011).

A novel and a rather unexpected approach to treatment of epilepsy have been recently proposed by (Sada, Lee, Katsu, Otsuki, & Inoue, 2015). These authors took their inspiration from the fact that some patients with drug‐resistant epilepsy benefit from a ketogenic diet which limits the intake of carbohydrates. Why this is beneficial is not known but the authors argue that it could be due to the impact on the “lactate shuttle” (Allaman, Belanger, & Magistretti, 2011; Mosienko, Teschemacher, & Kasparov, 2015; Pellerin and Magistretti 2003), whereby astrocytes supply lactate to the actively firing neurons to be used as energy substrate. Reduced supply of carbohydrates theoretically could limit utilization of glucose and therefore production of pyruvate and lactate by astrocytes in the brain. Sada et al. found that neural activity and seizures can be suppressed by lactate dehydrogenase (LDH) inhibition and suggested that LDH could be a target for treatment of epilepsy (see below).

Finally, astrocytic domain organization is disrupted in epilepsy which may, for example, affect K^+^ buffering or neurotransmitter clearance (Oberheim et al., 2008). Interestingly, wide‐spread reactive astrogliosis which develops in a mouse with a conditional deletion of β1‐integrin leads to spontaneous seizures, most likely due to the impaired uptake of glutamate (Robel et al., 2015). For further information on the role of astrocytes in epilepsy, see (Coulter and Steinhauser 2015; Robel 2016) .

#### 2.2.6 Ischemia/stroke

Stroke is one of the main causes of death worldwide and the leading cause of long‐term neurological disability. The only treatment with proven efficiency is thrombolysis by intravenous administration of recombinant tissue plasminogen activator. The role of astrocytes in stroke recently attracts more and more attention. Indeed, astrocytes are involved in a number of processes which profoundly influence tissue viability during and after ischemia. It is generally acknowledged that astrocytes are substantially more ischemia‐resistant than neurons and survive in conditions of limited blood supply, characteristic for penumbra surrounding the core of the ischemic infarction (Swanson, Farrell, & Stein, 1997; Vangeison, Carr, Federoff, & Rempe, 2008). These surviving astrocytes undergo activation and are involved in neuroprotection and post‐ischemic regeneration (Takano, Oberheim, Cotrina, & Nedergaard, 2009; Zhao and Rempe 2010). Astroglia contributions to brain resilience could include clearance of glutamate, control over K^+^ concentration, supply of lactate to the stressed neurons, secretion of neuroprotective factors, and scavenging reactive oxygen species by releasing GSH and ascorbic acid (Liu and Chopp 2015; Zhao and Rempe 2010). Recently, using optogenetic control of H^+^ pumps expressed on astrocytes, it was demonstrated that alkalinisation of astrocytes could reduce glutamate release and limit the ischemic brain damage in a cerebellar ischemia model. Therefore, controlling glial pH may be an effective therapeutic strategy (Beppu et al., 2014). In the absence of astroglia, the vulnerability of neurons to ischemia is greatly increased (Tanaka et al., 1999). Reactive astrocytes surrounding the ischemic core are the main contributors to the glial scar, along with oligodendrocytes and microglia, establishing a barrier between the damaged and surviving tissue. At the same time, astrocytes are involved in the pathology of stroke by production of neurotoxic substances, release of reactive oxygen species and by being a part of the brain edema mechanism (Liu and Chopp, 2015; Zhao and Rempe, 2010). After the stroke, scar formation and expression of proteoglycans might impede neurite outgrowth and inhibit structural and functional recovery (Cregg et al., 2014; Silver and Miller, 2004). Glial scars represent powerful barriers for re‐growth of axons, also in the case of mechanical trauma where astrogliosis is seen as a contributor to post‐traumatic epilepsy (Robel, 2016; Verellen and Cavazos, 2010). The triggers of glial transformation and activation in stroke or trauma remain elusive.

Thus, astrocytic processes may be either pathogenic in stroke/reperfusion or act as brain defense mechanisms which potentially could be harnessed for therapeutic benefits.

## 3 POTENTIAL THERAPEUTIC TARGETS IN ASTROCYTES

**Figure 1:**
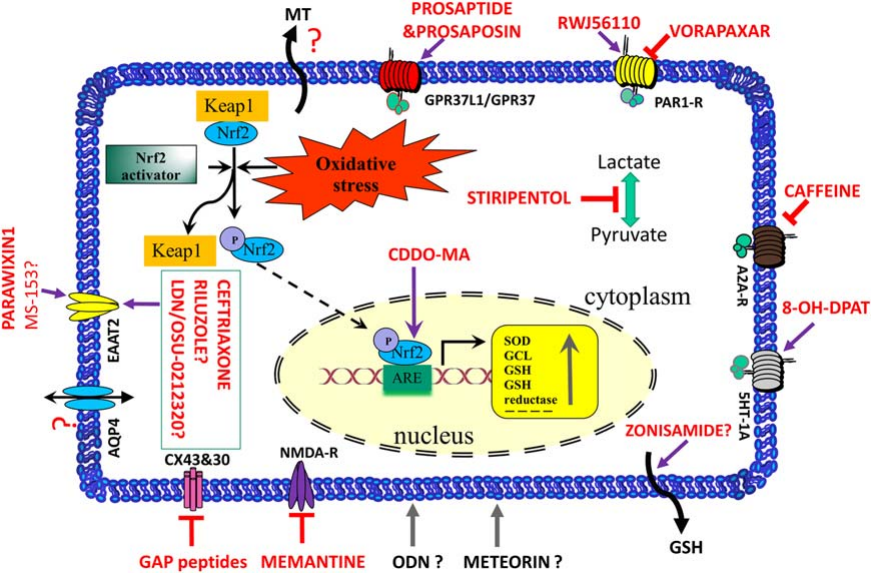
Major known neuroprotective pathways in astrocytes. Various pathways as discussed in the text demonstrate the multitude of potentially therapeutically exploitable neuroprotective mechanisms in astrocytes. Molecules which have been proposed as their activators or inhibitors are indicated in red (red arrows—putative activators; red T signs—putative inhibitors; red “?”—compounds with unclear mode of action). Metallothioneins (MT) and AQP4 water channels are also labeled with question mark signs since, so far, there is no established pharmacology for these pathways. For further details see the respective sections of the main text

Astrocytes possess a number of potentially targetable and therapeutically plausible biochemical or signaling pathways. In the following section, we summarize some of such candidate pathways and molecules and discuss their therapeutic potential. The key known neuroprotective pathways in astrocytes mentioned in this review are illustrated in Figure 1.

### 3.1 Glutamate transporters, glutamate transmission and excitotoxicity

As mentioned earlier, high concentrations of glutamate are neurotoxic. The most abundant glutamate transporter in the brain is EAAT2 (synonyms: GLT1 and SLC1A2) which is mainly expressed by astrocytes, making them a vital element of the defense against excitotoxicity (Fontana, 2015; Kim et al., 2011). Not surprisingly, loss or attenuation of glial glutamate transporters have been implicated in the pathogenesis of many CNS disorders, such as ALS (Rothstein, 2009), PD (Plaitakis and Shashidharan, 2000), stroke (Lai, Zhang, & Wang, 2014), epilepsy (Tanaka et al., 1997; Wetherington, Serrano, & Dingledine, 2008), HD (Arzberger, Krampfl, Leimgruber, & Weindl, 1997), AD (Jacob et al., 2007; Masliah, Alford, DeTeresa, Mallory, & Hansen, 1996), and major psychiatric disorders (Choudary et al., 2005; Lauriat and McInnes, 2007; Miguel‐Hidalgo et al., 2010). To the contrary, many animal studies indicate that upregulation of EAAT2 provides significant beneficial effects in models of disease (Harvey et al., 2011; Kong et al., 2012; Miller et al., 2012a; Takahashi et al., 2015b). Thus, EAAT2 represents a pharmacological target which may modify neuronal function or protect neurons.

The expression or activity of EAAT2 is regulated both transcriptionally and post‐transcriptionally (Grewer, Gameiro, & Rauen, 2014; Takahashi, Foster, & Lin, 2015a). Therefore, theoretically, upregulation of EAAT2 could be achieved at transcriptional or translational level. By screening of 1,040 FDA‐approved drugs and nutritionals, Rothstein et al. discovered some molecules which could increase transcription of the EAAT2 gene (Rothstein et al., 2005). The antibiotic ceftriaxone is one of the best‐studied candidates amongst this group, it has the longest half‐life of available ß‐lactam antibiotics and is believed to penetrate blood brain barrier (Yogev, Shulman, Chadwick, Davis, & Glogowski, 1986). Ceftriaxone reduces glutamate excitotoxicity in animal models of PD, HD, ischemia, and multiple sclerosis (Cudkowicz et al., 2014; Hu et al., 2015; Kelsey and Neville, 2014; Miller et al., 2008). Ceftriaxone also delays loss of neurons and prolongs survival in mouse models of amyotrophic lateral sclerosis and stroke (Guo et al., 2003; Thone‐Reineke et al., 2008). In a clinical trial where ceftriaxone was tested for treatment of ALS patients, it was well tolerated in stages I and II (Berry et al., 2013). Unfortunately, stage III was discontinued because no increase of the length of survival or prevention of a functional decline was achieved (Cudkowicz et al., 2014). However, it may still be possible to develop derivatives of ceftriaxone with improved properties. It is also possible that ALS was not the best disease target for it.

Currently, riluzole is the only FDA‐approved drug for the treatment of ALS, although it prolongs the life of ALS patients by only 7 months (Miller, Mitchell, & Moore, 2012b). A major action of riluzole is the inhibition of glutamate release from presynaptic neurons, but it also enhances astrocytic glutamate uptake by upregulating EAAT2 gene expression (Liu et al., 2011).

Colton et al. developed a cell‐based enzyme‐linked immunosorbent assay approach to search for translational enhancers and identified 61 compound which increased EAAT2 protein levels (Colton et al., 2010). These compounds enhanced glutamate transport without changing EAAT2 mRNA level (Colton et al., 2010). The same group developed thiopyridazine and pyridazine derivatives that increase EAAT2 expression (Xing et al., 2011). Analog LDN/OSU‐0212320, a pyridazine derivative, protected cultured neurons from glutamate‐mediated excitotoxic injury. It also delayed motor function decline and extended lifespan in an animal model of ALS (Kong et al., 2014). Further tests of this analog in a range of animal models will potentially reveal other diseases where reduction of excessive extracellular glutamate can provide therapeutic advantage. Wider testing of these compounds in other models and species but mice should verify the therapeutic potential of this strategy.

In addition to EAAT2 transcriptional and translational activators, there are chemicals that directly modulate the function of EAAT2. Parawixin1, purified from the venom of the spider *parawixia bistriata*, enhances directly and selectively EAAT2 function by facilitating conformational transitions involved in substrate translocation (Fontana et al., 2007). Site‐directed mutagenesis identified a structural region within EAAT2 which is important for the transporter‐enhancing activity in transmembrane domains 2, 5, and 8 (Mortensen, Liberato, Coutinho‐Netto, Dos Santos, & Fontana, 2015). This unique structural information could be employed in hybrid structure‐based virtual screening of a large library to identify novel allosteric modulators of EAAT2. Another EAAT2 activator is the pyrazoline compound MS‐153 ([R]‐5‐methyl‐1‐nicotinoyl‐2‐pyrazoline) (Shimada et al., 1999) although recently it has been questioned whether its effects are actually attributable to action on EAAT2 or are a consequence of other effects such as inhibition of Ca^2+^ channels.

### 3.2 GSH

Decreased brain content of GSH is an indicator of oxidative stress which, in turn, is recognized as a central contributing factor to neurodegenerative diseases (Kim et al., 2015). Although GSH can cross the blood‐brain barrier, blood is probably not the major source of cerebral GSH (Anderson, Underwood, Bridges, & Meister, 1989). Instead, the predominant source in the brain is astrocytes, and this allows neurons to maintain a sufficient antioxidant defense. Hence upregulating astrocytic GSH production could be a potential neuroprotective strategy. Zonisamide, a novel anti‐PD agent used in Japan, increased GSH levels in the striatal astrocytes and demonstrated neuroprotective effects against dopaminergic neurodegeneration in PD mice (Asanuma et al., 2010). However, this drug upregulates expression of a whole range of factors which are also potentially neuroprotective and neurotrophic.

### 3.3 Metallothioneins

Metallothioneins (MT) are a family of low molecular weight and cysteine‐rich proteins with antioxidant, anti‐apoptotic, and anti‐inflammatory properties (Bolognin, Cozzi, Zambenedetti, & Zatta, 2014). MT has been implicated in neurodegenerative diseases including PD, AD, and also brain trauma and ischemia (Hozumi, 2013). Neuroprotective properties of MT are well documented (Chung, Hidalgo, & West, 2008; Vasak, 2005). Deficiency in MT generally worsens the damage caused by neurotoxic factors or trauma (Giralt et al., 2002). However, in *glioblastoma multiforme* patients, high levels of MT are a negative prognostic factor, probably because MT make tumors more resistant to therapy (Mehrian‐Shai et al., 2015).

The MT family is comprised of four main members, MT1 to MT4. MT1 and MT2 are primarily expressed in astrocytes and it is thought that astrocyte‐derived MT facilitate neuronal survival and axonal regeneration (Aschner, 1997; Hidalgo, Aschner, Zatta, & Vasak, 2001). Exogenous MT1 and MT2 improved neuronal survival and axonal outgrowth in cortical, hippocampal, and dopaminergic cultures (Chung, Vickers, Chuah, & West, 2003), and astrocytic MT protected dopaminergic neurons in a PD model (Miyazaki et al., 2011). In contrast, MT1 and MT2 double knockout mice demonstrated impaired axonal regeneration after sciatic nerve crush and MT2A treatment promoted neurite elongation and post‐injury reactive neurite growth (Chung et al., 2003).

The exact mechanism of MT‐mediated neuroprotection is not known but possibly it involves zinc‐mediated transcriptional activation of genes involved in growth, proliferation, and differentiation (Sharma and Ebadi, 2014; Sharma, Rais, Sandhu, Nel, & Ebadi, 2013). MT also regulates copper metabolism and potentially by this route MT1 overexpression can slow disease progression in SOD1 (G93A) mice (model of ALS) (Tokuda, Okawa, Watanabe, & Ono, 2014). MT also reduce oxidative damage (Bolognin et al., 2014; Uttara, Singh, Zamboni, & Mahajan, 2009).

Interestingly, ageing is often accompanied by various late‐life neurodegenerative diseases, while MT show strong anti‐ageing effects (Sharma and Ebadi, 2014). Dietary supplements combined with genetically increased MT1 have been demonstrated to increase lifespan in mice (Yang et al., 2006). Interestingly, exercise induces MT, at least in the spinal cord (Hashimoto, Hayashi, Inuzuka, & Hozumi, 2009). So far, no pharmacological compounds have been reported to specifically induce MT synthesis in astrocytes or non‐selectively in the brain. Nevertheless, given the example of EAAT2 inducers (see above), this does not look like an implausible idea.

### 3.4 Aquaporin 4

The AQP4 water channel is exclusively expressed by astrocytes and constitutes an astrocyte‐specific mechanism regulating fluid homeostasis which is fundamental for brain function (Badaut, Lasbennes, Magistretti, & Regli, 2002; Nielsen et al., 1997). The enrichment of AQP4 in astroglial endfeet surrounding blood vessels suggests that it regulates not only astrocyte volume, but also the water traffic between vascular and interstitial compartments, as well as the size, shape and diffusion characteristics of the extracellular space (Xiao and Hu, 2014). AQP4 is co‐localized with Kir4.1, indicating that coordinated action of both channels is required to maintain K^+^ homeostasis (Masaki et al., 2010). Neuronal activity leads to transient increases in the extracellular K^+^ concentration and clearance of the excess K^+^ from the extracellular space is an important function of astrocytes.

AQP4 knockout mice (both non‐selective and glia‐targeted) have a significantly reduced tendency to develop cerebral edema following water intoxication and stroke, as well as better survival and neurological outcomes (Haj‐Yasein et al., 2011b; Manley et al., 2000). Given the role of AQP4 in K^+^ and water homeostasis, it seems rational to develop AQP4 modulators as drugs against diseases involving brain edema (King, Yasui, & Agre, 2000). Unfortunately, limited progress has been made in AQP4‐targeted therapeutics (Verkman, Anderson, & Papadopoulos, 2014). This is partly due to the lack of robust assays of AQP4 activity. The small size of the functional AQP4 monomer and its very small pore diameter, which prevents the access of conventional small molecules, translates to poor “druggability” (Verkman et al., 2014). As AQP4 are simple passive pores, they lack sophisticated gating and transport mechanisms suitable for targeting with small molecules. Furthermore, mutations in the extracellular and cytoplasmic domains of AQP4 generally have little effect on water permeability through the channel, which suggests that the binding of an inhibitor has to occur deep in the narrow pore to physically prevent water conduction (Papadopoulos and Verkman, 2013; Verkman et al., 2014). Nevertheless, further large‐scale screening of random and computationally biased libraries in search of AQP4 blockers is warranted. Rigorous tests for validation of putative lead compounds also need to be developed.

### 3.5 Connexin gap junctions

In contrast to most mature neurons, astrocytes are usually coupled through gap junctions (GJ) to form large intercellular networks (Rouach, Glowinski, & Giaume, 2000). GJ channels are built of connexin (CX) proteins, of which CX43 and CX30 are the major subtypes in astrocytes (Giaume and McCarthy, 1996). Individual CX assemble into hexamers to form transmembrane channels, termed connexons, which couple with apposing connexons on neighboring cells. Dense GJ plaques may contain thousands of channels (Unwin and Zampighi, 1980). GJ couple the cytoplasm of connected cells and permit movement of ions and low molecular weight molecules (about 1–2 kDa (Loewenstein, 1981).We still do not know to what extent selectivity of the GJ can change under different circumstances.

GJ between astrocytes allow movement of metabolic substrates and support astrocytic spatial K^+^ buffering to modulate and potentially synchronize neuronal activity (Gardner‐Medwin, 1983). GJ are also dense at the endfeet of astrocytes where they provide a perivascular route that facilitates intercellular trafficking between neighboring endfeet (Simard, Arcuino, Takano, Liu, & Nedergaard, 2003). Connexons exist also on their own as single membrane hemichannels which connect the cell cytoplasm to the extracellular milieu (Giaume, Leybaert, Naus, & Saez, 2013). It is now known that under certain conditions CX hemichannels can release ATP (Huckstepp et al., 2010) or lactate (Karagiannis et al., 2016).

Intercellular communication among astrocytes is lost in CX43/CX30 double knockout mice, demonstrating their pivotal role in astroglial connectivity (Dermietzel et al., 2000; Giaume and Theis 2010). CX43/CX30 double knockout leads to impaired potassium clearance and disrupts synaptic transmission and plasticity (Pannasch et al., 2011; Wallraff et al., 2006). CX43/CX30 double knockout also causes astrocyte endfeet edema and weakens the blood‐brain barrier (Ezan et al., 2012). Under pathological conditions, altered CX expression may lead to a failure of glial communication (Rouach et al., 2002). Changes in CX43 expression have been detected in animal models and human patients with epilepsy, in ischemia and stroke, autism and neurodegenerative diseases (Takeuchi and Suzumura 2014).

Specific small molecule modulators of CX43/CX30 are not available and, at present, the best‐characterized tools to target specific CX are peptides that mimic a short stretch of amino acids on the extracellular loop motifs of the target connexons. These interfere with GJ formation and inhibit hemichannel activity (Evans and Boitano, 2001; Leybaert et al., 2003). Because their initial characterization (Dahl, Nonner, & Werner, 1994), a series of “Gap” peptides with specificity for certain CX were developed (Abudara et al., 2014; Chaytor, Evans, & Griffith, 1997; Evans and Boitano 2001; Gomes, Srinivas, Van Driessche, Vereecke, & Himpens, 2005; Leybaert et al., 2003). A promising new report shows that Gap19, a nonapeptide derived from the cytoplasmic loop of CX43, inhibits astroglial CX43 hemichannels, while not affecting GJ channels (Abudara et al., 2014). Moreover, Gap19 is specific to CX43 and was demonstrated to cross the blood–brain barrier when coupled to the HIV‐derived TAT internalization sequence (Abudara et al., 2014). The effect of this peptide in neuroprotection is currently being explored (Freitas‐Andrade and Naus, 2016).

Because of their importance for astrocytic functions, CX30 and CX43 represent potential drug targets of interest, although further studies are needed in order to understand the precise molecular mechanism regulating their gating properties. However, development of small molecules for such targets clearly requires thinking out of the box.

### 3.6 PAR‐1 receptors

Protease‐activated receptors (PAR) are G‐protein‐coupled receptors (GPCR) activated by extracellular serine proteases. The thrombin receptors PAR‐1, −3 and −4 and the tryptase/trypsin receptor PAR‐2 are abundant in CNS (Ramachandran, Noorbakhsh, Defea, & Hollenberg, 2012). PAR are characterized by the presence of a tethered peptide ligand in their N‐terminal part which, when released by cleavage, acts on the ligand binding site and activates the receptor. The expression of PAR in the brain is differentially regulated in neurodegenerative disorders like PD, AD, multiple sclerosis and stroke (Luo, Wang, & Reiser, 2007). Activation of PAR can lead to cell death or cell survival, depending on the magnitude and the duration of agonist stimulation.

PAR‐1 is the best characterized receptor of this family which is activated by the cleavage of the extracellular N‐terminal domain by thrombin. This releases a tethered ligand (SFLLRN) that activates the receptor and initiates signaling through *G*_q/11_, *G*_i/o_, or *G*_12/13_ G‐proteins (Coughlin 2000; Traynelis and Trejo, 2007). In the CNS, PAR‐1 is expressed mainly (and in some areas almost exclusively) by astrocytes although in the hippocampus PAR‐1 is also present in some neurons (Junge et al., 2004; Niclou, Suidan, Brown‐Luedi, & Monard, 1994; Wang, Ubl, & Reiser, 2002). Activation of PAR‐1 leads to increases in astrocytic [Ca^2+^]_i_ and astrocytes activated by PAR‐1 agonists can release glutamate which, in turn, may activate NMDA receptors on adjacent neurons (Lee et al., 2007; Vance, Rogers, & Hermann, 2015).

Activation of PAR‐1 might produce bimodal effects. Low‐level PAR‐1 activation seems to be protective whereas high levels of PAR‐1 activation compromise cell viability (Acharjee et al., 2011; Donovan, Pike, Cotman, & Cunningham, 1997; Vaughan, Pike, Cotman, & Cunningham, 1995). The neuroprotective effects of thrombin via PAR‐1 activation have been confirmed in several independent studies both *in vitro* and *in vivo*. PAR‐1 activation protected neurons and astrocytes against chemical insults, via regulation of the secretion of cytokine‐induced neutrophil chemoattractants (Wang, Luo, & Reiser, 2007). PAR‐1 activation by thrombin, further, diminished ceramide‐induced astrocyte death via upregulation of JUN N‐terminal kinase (Wang, Luo, Stricker, & Reiser, 2006), and rescued astrocytes through the PI3K/Akt signaling pathway from chemically induced apoptosis (Zhu and Reiser, 2014). *In vivo* PAR‐1 activation was neuroprotective in a 6‐hydroxydopamine model of PD (Cannon et al., 2006).

In contrast, a number of studies suggest a pathophysiological role for PAR‐1 in various types of brain damage (Gutierrez‐Rodriguez and Herranz, 2015). In a murine model of stroke, neurotrauma and brain hemorrhage, PAR‐1‐mediated signaling had deleterious effects on neuronal survival and function. PAR‐1 deficiency or its pharmacological inhibition with an antagonist BMS‐200261 reduced infarct volume in the transient occlusion of the middle cerebral artery model (Junge et al., 2003). PAR‐1 deficiency or the central application of PAR‐1 antagonists also reduced neuronal injury following intrastriatal injection of NMDA in rats (Hamill, Mannaioni, Lyuboslavsky, Sastre, & Traynelis, 2009). PAR‐1 inhibitors reduced brain damage caused by the neurotoxic effects of blood in intracerebral hemorrhage (Xue, Hollenberg, Demchuk, & Yong, 2009). PAR‐1 could also be involved in the pathogenesis of chronic neurodegenerative and/or inflammatory conditions. Post‐mortem tissue samples from patients affected by HIV‐associated dementia, a neurodegenerative condition affecting patients with AIDS, show that PAR‐1 expression is enhanced in astrocytes, which in turn could induce expression of inflammatory mediators by these cells (Acharjee et al., 2011; Boven et al., 2003). PAR‐1 deficiency, as well as the intraventricular administration of PAR‐1 antagonists, also reduced dopaminergic neuron damage and microgliosis in a MPTP model of PD (Hamill et al., 2007). Possibly, reports of a potential pathogenic role of PAR‐1 signaling can be linked to facilitation of glutamate release activation of NMDA receptors as mentioned above.

Importantly, there are already small molecule PAR‐1 antagonists such as vorapaxar, also known as Zontivity, marketed as anti‐platelet drug (Bhandari and Mehta, 2014) and other prototype molecules such as RWJ‐56110 (Andrade‐Gordon et al., 1999).

To summarize, PAR‐1 modulation may be seen as a fairly astrocyte‐specific intervention within the brain. However, a major concern with the systemic use of PAR‐1 antagonists is their anti‐thrombotic effect. Therefore, a centrally acting drug which modulates astrocytic PAR‐1 would need to be devoid of hematinic side effects, which may be achievable using a pro‐drug strategy.

### 3.7 Astrocytic GPR37 and GPR37L1

GPR37 and GRP37L1 are two closely related GPCRs which are almost exclusively expressed in CNS in mammals. GPR37 is alternatively known as the Parkin‐Associated Endothelin‐Like receptor (Pael‐R) (Imai et al., 2001), while GP37L1 is named for its similarity to GPR37. The interest to GPR37 was boosted by its potential link to PD. Parkin is an E3 ubiquitin‐protein ligase and mutations in this gene are directly linked to autosomal recessive juvenile PD (AR‐JP). Although parkin has many substrates, GPR37 attracted interest because GPR37 is up‐regulated in brains of AR‐JP patients (Takahahshi, 2006). In addition, GPR37 is present in the core of Lewy bodies, thus suggesting a role of GPR37 aggregates in PD. Also, viral vector‐mediated GPR37 overexpression in substantia nigra results in progressive degeneration of nigral dopaminergic neurons (Dusonchet, Bensadoun, Schneider, & Aebischer, 2009; Low and Aebischer, 2012).

Under normal conditions, correctly folded GPR37 is trafficked to the cell surface but it has a high propensity to misfold. Parkin ubiquitinates misfolded GPR37 targeting it for proteasomal degradation. If this process fails, misfolded GPR37 forms aggregates. Mutations in the parkin gene enhanced dopaminergic neuronal cytotoxicity by failing to remove aggregated GPR37 and other substrates. This leads to activation of the unfolded protein response and cell death, a process that can be rescued by re‐expression or overexpression of wild type parkin (Imai et al., 2001). GPR37L1 does not undergo ubiquitination and thus the phenomenon is limited to GPR37.

Up until relatively recently, the physiological functions of GPR37 and GPR37L1 were assessed mainly through use of knockout mice. The connection between GPR37 and parkin has led to a focus on the dopaminergic system in GPR37 knockout mice which exhibit progressive loss of dopaminergic neurons, various subtle alterations to dopaminergic signaling and significantly reduced locomotor activity (Marazziti et al., 2011, 2004, 2007). In humans, dysregulation of GPR37 has recently been linked to major depressive disorder, bipolar disorder and autism spectrum disorder (Cruceanu et al., 2015; Fujita‐Jimbo et al., 2012; Tomita et al., 2013). In contrast to GPR37, the phenotype of GPR37L1 knockout mice is less well characterized. The most remarkable observation in these animals is that they are hypertensive (Min et al., 2010) and have cardiac hypertrophy probably due to hypertension (Min et al., 2010). The link between GPR37L1 and blood pressure control remains elusive. Another study reported abnormal cerebellum development in GPR37L1 knockout mice that was a direct consequence of premature downregulation of granule neuron precursor cell proliferation and concomitant premature development and maturation of Bergmann glia and Purkinje neurons (Marazziti et al., 2013).

Deorphanisation of GPR37 and GPR37L1 had been a difficult process. Although they were originally identified through searches for homologs of endothelin and bombesin receptors, neither GPR37 nor GPR37L1 bind endothelins or related peptides (Leng, Gu, Simerly, & Spindel, 1999; Zeng, Su, Kyaw, & Li, 1997). Eventually, an extracellular peptide, prosaposin, and its active peptide fragments, prosaptides (including the synthetic analog TX14A), were identified as agonists of GPR37 and GPR37L1 (Meyer, Giddens, Schaefer, & Hall, 2013). Both prosaposin and prosaptides have long been known as powerful and essential neuroprotective and glioprotective factors (O'Brien et al., 1995; Obrien, Carson, Seo, Hiraiwa, & Kishimoto, 1994). Mutations in prosaposin in mammals result in severe neurodegeneration (Sikora, Harzer, & Elleder, 2007; Yoneshige, Suzuki, Suzuki, & Matsuda, 2010). Prosaposin and prosaptides were shown to couple via Gαi and Gαo proteins which are pertussis toxin‐sensitive (Hiraiwa, Campana, Martin, & O'Brien, 1997; Yan, Otero, Hiraiwa, & O'Brien, 2000). The peptides interacted with, at the time, unknown receptors with nanomolar affinity and stimulated ERK phosphorylation (Subramaniam and Unsicker, 2010). Indeed, recently Meyer and colleagues found that GPR37 and GPR37L1 met all these previously established characteristics (Meyer et al., 2013).

Within the brain, GPR37 mRNA was detected both in neurons and glia (Zeng et al., 1997) but it seems that only some neuronal types such as dopaminergic (and probably other catecholaminergic neurons) express it at high level. In contrast to the mixed distribution of GPR37, GPR37L1 is highly expressed in astrocytes, with in situ hybridization revealing greatest density of GPR37L1 within the Bergmann glia of the cerebellum (Valdenaire et al., 1998). Microarray studies reported more than 100 times higher expression of GPR37L1 in rat and mice astrocytes compared with neurons (Cahoy et al., 2008; Lovatt et al., 2007; Zhang et al., 2014). GPR37L1 is also expressed in oligodendrocytes (Zhang et al., 2014). These results are consistent with our own, yet unpublished, transcriptomic analysis of rat brainstem astrocytes.

Given that the ligands of these receptors have well established neuroprotective activities and that GPR37 and GPR37L1 are highly expressed in astrocytes, one may speculate that the beneficial effects of prosaposin and its derivatives might be mediated at least partially by astrocytes rather than by a direct action on the neurons. Prosaptide acting on GPR37/GPR37L1 clearly protected cultured astrocytes from oxidative stress (Meyer et al., 2013). One important direction of current research is to assess how these two receptors regulate astrocytic function and, via this route, modulate activity and survival of neurons. An important question is also whether these effects are specific to only some subtypes of neurons, for example catecholaminergic neurons. Currently, no small molecule ligands for either GPR37 or GPR37L1 are available, and thus the pharmacology of these receptors is unexplored terrain that has the potential to yield clinically useful therapeutic drugs. If any of the *in vivo* protective effects of prosaposin are indeed dependent on astrocytic GPR37and/or GPR37L1, then a screen for small molecule agonists and/or positive allosteric modulators for these receptors would be warranted. Such compounds may have outstanding therapeutic value due to their potential to mimic and/or enhance the glio‐ and neuroprotective actions of secreted prosaposin.

### 3.8 Targeting astrocytic adenosine receptor A2a to improve memory in AD

Adenosine is a potent neuromodulator derived from breakdown of ATP and other adenine nucleotides. Adenosine and ATP are released in the brain by diverse cell types (Burnstock, 2007). A1 and A3 are Gi‐coupled, while A2A and A2B are Gs‐coupled receptors which inhibit and trigger, respectively, cyclic AMP (cAMP)‐mediated signaling. A2A receptors are highly expressed in the brain and have been implicated in diverse neuropathologies, including PD, ischemic brain injury, traumatic brain injury and schizophrenia (Chen et al., 2007; Matos et al., 2015). A2A receptors on glial cells and their impact on the neuroinflammatory and neuromodulatory processes are likely to be involved in these diseases. Indeed, the A2A receptor regulates astrocytic functions (Matos, Augusto, Agostinho, Cunha, & Chen, 2013) and has been implicated in AD (Albasanz, Perez, Barrachina, Ferrer, & Martin, 2008; Huang and Mucke, 2012). Astrocytic A2A receptors seem to affect the ability of Aβ peptide to suppress glutamate uptake, which could be one of the mechanisms of excitotoxicity in AD (Matos et al., 2012). Although microglia also expresses A2A receptors, increased levels of A2A receptor expression in humans with AD are found only in astrocytes. Similar to AD humans, aging mice expressing human amyloid precursor protein also have increased levels of astrocytic A2A receptors (Orr et al., 2015). Conditional genetic removal of these receptors enhanced memory in these mice, suggesting that inhibiting astrocytic A2A receptors might be considered as a therapeutic strategy for memory enhancement. In line with this speculation, there has been some evidence showing that caffeine, whose main target is A2A receptors, can improve normal memory function or even prevent AD symptoms in older adults (Arendash and Cao, 2010; Borota et al., 2014; Carman, Dacks, Lane, Shineman, & Fillit, 2014). However, the case is not clear, because deletion of astrocytic A2A receptors disrupts glutamate homeostasis, leading to psychomotor and cognitive impairments which resemble schizophrenia (Matos et al., 2015).

### 3.9 Meteorin pathway

Meteorin was first identified as a retinoic‐acid‐responding gene involved in glial differentiation and regulation of axonal extension (Nishino et al., 2004). It is a fairly long peptide ‐ 291 amino acids in the mouse, including a 21 amino acid signaling peptide. Meteorin is mainly produced and secreted by astroglia and, in addition to the effects on glia and neurons, also acts on endothelial cells (Park et al., 2008). Lentiviral overexpression of meteorin protected striatal neurons from excitotoxicity caused by quinolinic acid *in vivo* (Jorgensen et al., 2011) and reversed hypersensitivity in rat models of neuropathic pain (Jorgensen et al., 2012). Meteorin is upregulated in reactive astrocytes in a photothrombotic ischemia mouse model and functions as a negative feedback effector in reactive gliosis (Lee et al., 2015). However, the cellular receptor(s) for meteorin are still unknown. It has been reported that meteorin acts through the Jak‐STAT3 pathway to promote glial differentiation in neural stem cells (Lee, Han, Lee, Park, & Kim, 2010). However, exogenous treatment of astrocytes with meteorin did not activate the same pathway (Lee et al., 2015). This might be due to the existence of more than one meteorin receptor, with different signaling mechanisms. Nevertheless, once identified, this receptor may become an interesting therapeutic target for neuroprotection.

### 3.10 Metabotropic octadecaneuropeptide (ODN) receptor

The CNS is sensitive to oxidative stress due to its high metabolic rate and high levels of unsaturated lipids. ODN is a peptide (QATVGDVNTDRPGLLDLK) generated through the proteolytic cleavage of the 86‐amino acid precursor protein “diazepam‐binding inhibitor” which is expressed by astrocytes (Burgi, Lichtensteiger, Lauber, & Schlumpf, 1999; Malagon et al., 1993), although probably not completely exclusively (Alho, Harjuntausta, Schultz, Pelto‐Huikko, & Bovolin, 1991). ODN is a potent protective agent that prevents oxidative stress‐induced apoptosis and attenuates H_2_O_2_‐evoked inhibition of SOD and catalase activities in astrocytes (Hamdi et al., 2011). It has been suggested that the anti‐apoptotic activity of ODN is mediated through a putative GPCR coupled to the adenylate cyclase/protein kinase A pathway (Hamdi et al., 2012). Downstream of protein kinase A, ODN induces ERK phosphorylation which, in turn, activates the expression of the anti‐apoptotic gene Bcl‐2 and blocks the stimulation by H_2_O_2_ of the proapoptotic gene Bax. The effect of ODN on the Bax/Bcl‐2 balance could possibly explain its antagonism of the deleterious action of H_2_O_2_ on mitochondrial membrane integrity and caspase‐3 activation (Hamdi et al., 2012, 2015). This anti‐apoptotic effect of ODN might be important in neurodegenerative diseases and stroke. If a dedicated GPCR for ODN exists, it could be yet another potential candidate for the development of small molecules agonists to be used for the treatment of ischemia and neurodegenerative diseases.

### 3.11 Serotonin 1A receptors on astrocytes as a potential route for treatment of PD

The 5‐HT1A receptor, one of 14 subtypes of metabotropic receptors for serotonin, is widely distributed in brain (Barnes and Sharp, 1999). As a key mediator of serotonergic signaling in the CNS, the 5‐HT1A receptor is involved in numerous effects of central serotonin, ranging from cognition and emotion control to neurite outgrowth and synapse formation (Filip and Bader, 2009; Ohno, 2011; Pucadyil, Kalipatnapu, & Chattopadhyay, 2005).

Quite commonly, effects mediated through 5‐HT1A receptors are claimed to be mediated by neurons. However, since a very long time it has been known that astrocytes also express serotonin receptors and respond to serotonin with increases in [Ca^2+^]_i_. Several studies pointed at the therapeutic potential of astrocytic 5‐HT1A receptors. Stimulation of 5‐HT1A receptors on astrocytes promotes astrocyte proliferation and neuroprotection both *in vitro* and in PD model mice (Miyazaki et al., 2013). The 8‐OH‐DPAT [(R)‐(+)−8‐hydroxy‐2‐(di‐n‐propylamino)tetralin hydrobromide], a full 5‐HT1A agonist, enhances astrocyte proliferation in mouse striatum. The 8‐OH‐DPAT significantly up‐regulates astrocytic antioxidant pathways by increasing the expression of erythroid 2‐related factor 2 (Nrf2) (Miyazaki et al., 2013) which activates genes involved in anti‐oxidant defense (see below). Nrf2‐regulated genes are preferentially activated in astrocytes, boosting their detoxification and antioxidant functions (Vargas and Johnson, 2009). Activation of Nrf2 in astrocytes protects dopaminergic neurons from oxidative stress (Miyazaki et al., 2011; Wong et al., 2003). Protein S100ß is expressed in various cell types with the highest level in the cytoplasm of astrocytes (Selinfreund, Barger, Pledger, & Vaneldik, 1991) which release it into the extracellular space. Extracellular S100β has autocrine effects and promotes astrocytic proliferation (Donato, 2003). Stimulation of 5‐HT1A receptors on astrocytes leads to secretion of S100β which seems to be protective at nanomolar concentrations although deleterious at micromolar concentrations. It is therefore conceivable that pharmacological modulation of 5‐HT1A receptors on astrocytes could be astro‐ and neuroprotective [for more detail see (Miyazaki and Asanuma, 2016)].

### 3.12 Targeting of astrocytic LDH enzymes to treat epilepsy

In addition to glucose, lactate is a major source of energy in the brain, and a significant amount of lactate is produced through glycolysis by astrocytes (Dienel, 2012; Gladden, 2004). LDH catalyzes the interconversion of pyruvate and lactate; some of which is transported from astrocytes to neurons via the so called “lactate shuttle” (Chih and Roberts Jr., 2003; Pellerin and Magistretti, 1994). In addition, lactate may have a signaling role in the brain (Tang et al., 2014), see also our recent review (Mosienko et al., 2015).

In epilepsy where activity of hyperexcitable neurons is uncontrollably synchronized, abundant energy for these activities has to be supplied (Bertram, Zhang, Mangan, Fountain, & Rempe, 1998). Expectedly, high rates of glucose metabolism and elevated activity of LDH have been shown in human epilepsy and in animal models (Dufour, Koning, & Nehlig, 2003). A recent study suggested that the effectiveness of a ketogenic diet against epilepsy is linked to bypassing glycolysis in astrocytes (Sada et al., 2015). It was found that that inhibition of LDH hyperpolarised neurons, reducing their excitability, and that this could be reversed by pyruvate, which supports the notion of it being a metabolic, rather than receptor mediated action. It turned out that stiripentol, which is sometimes used to treat epilepsy, is an LDH inhibitor. Moreover, an analog of stiripentol was found which proved to be effective *in vivo* in a rodent epilepsy model, thus potentially setting up a new class of anti‐epileptic therapies (Sada et al., 2015). Other findings also implicate lactate in epilepsy, for example, altered level and cellular distribution of monocarboxylate transporters (Perez et al., 2012).

In contrast, some studies suggested that lactate can be neuroprotective (Jourdain et al., 2016; Lee et al., 2012).Therefore, reduction of lactate production by LDH inhibitors is a double‐edged sword strategy since compromising neuroprotection is undesirable. This issue requires further exploration using new models and, perhaps, other species but mice.

### 3.13 Nrf2‐ARE pathway

Maintaining redox homeostasis in the brain is essential for survival. One critical pathway through which the cell regulates its antioxidant defense is the Nrf2‐antioxidant response element (ARE) (Johnson and Johnson, 2015) which is a cis‐acting regulatory element controlling expression of phase II detoxifying and antioxidant genes (Rushmore, Morton, & Pickett, 1991; Rushmore and Pickett, 1990). Nrf2 is a cytoplasmic protein sequestered by actin‐bound protein Keap1 (Kelch ECH associating protein) (Itoh et al., 1999; Zipper and Mulcahy, 2002). Under normal unstressed conditions, Nrf2 is anchored to Keap1 and rapidly degraded (Itoh et al., 2003; McMahon, Itoh, Yamamoto, & Hayes, 2003). This process seems to be much more powerful in neurons than in astrocytes (Jimenez‐Blasco, Santofimia‐Castano, Gonzalez, Almeida, & Bolanos, 2015). Oxidative stress or exposure to electrophilic agents that react with Keap1 slow down Nrf2 degradation and lead to its nuclear accumulation. Nrf2 binding to the ARE drives expression of several detoxifying and antioxidant genes including SOD, GCL, GSH synthase, GSH peroxidase, GSH reductase and γ‐glutamine cysteine synthase, boosting anti‐oxidant defence (Kensler, Wakabayash, & Biswal, 2007; Sykiotis and Bohmann, 2010). Hence, the Nrf2‐ARE pathway is considered a high‐value therapeutic target (de Vries et al., 2008; Johnson and Johnson, 2015; van Muiswinkel and Kuiperij, 2005). It is preferentially activated in astrocytes while neurons largely depend on astrocytes for the antioxidant defense (Kraft, Johnson, & Johnson, 2004; Lee, Calkins, Chan, Kan, & Johnson, 2003; Shih et al., 2003). Therefore, unsurprisingly, many studies report that activation of the Nrf2 pathway in astrocytes is neuroprotective (Calkins, Vargas, Johnson, & Johnson, 2010; Chen et al., 2009; Gan, Vargas, Johnson, & Johnson, 2012; Vargas, Johnson, Sirkis, Messing, & Johnson, 2008). For example, astrocyte‐specific overexpression of Nrf2 protects dopaminergic neurons in MPTP‐injected Nrf2‐deficient parkinsonic mice (Chen et al., 2009). For further information see (Buendia et al., 2016; Joshi and Johnson, 2012).

Numerous cell‐based and *in silico* screens have identified Nrf2‐activating compounds (Schaap, Hancock, Wilderspin, & Wells, 2013; Wang et al., 2013; Williamson et al., 2012; Wu, McDonald, Liu, Chaguturu, & Klaassen, 2012), including triterpenoid 2‐cyano‐3,12‐dioxooleana‐1,9(11)‐dien‐28‐oate‐methylamide (CDDO‐MA), puerarin, sulforaphane, CDDO‐ethyl amide and others. Nrf2 activators demonstrated activity in *in vitro* and *in vivo* in different neurodegenerative mouse models, protecting neurons, decreasing the accumulation of aberrant proteins and increasing life span (Buendia et al., 2016; Joshi and Johnson 2012). The existing data are strongest for PD, ALS, and multiple sclerosis models, but the therapeutic potential of this pathway in AD and HD is under investigation. In conclusion, the Nrf2–ARE pathway is definitely a promising target in neurodegenerative diseases with several classes of small molecules already demonstrated to act as its inducers.

## 4 CONCLUDING REMARKS

The mechanisms and pathologies mentioned in this review by no means exhaust the list of known astroglial neuroprotective or therapeutic mechanisms. For example, astrocytes could be an important target for antidepressants which block re‐uptake of noradrenaline (Hertz, Chen, Gibbs, Zang, & Peng, 2004) and there is evidence that statins can reduce release of APOE from astrocytes (Naidu, Xu, Catalano, & Cordell, 2002). Only about 30 years ago, the very thought that a centrally acting drug may target an astrocytic receptor seemed implausible. For instance, monoamine oxidase B (MAO‐B) which is a target for the antidepressant deprenil and is localized almost exclusively in astrocytes (Riederer et al., 1987), has recently attracted attention because it can be used as an activator for pro‐drugs that, after the reaction with MAO‐B, become cytotoxic for glioma cells, which typically upregulate MAO‐B (Sharpe and Baskin, 2016). Irrespective of glioma treatment, MAO‐B potentially could be used for local activation of other astroglia‐targeted molecules.

To sum up, the neurocentric view of brain function and disease has been challenged by extensive data supporting the physiopathological and therapeutic potential of astroglia. A solid body of evidence now indicates that harnessing the natural capacity of astrocytes to protect neurons is a promising clinical strategy. Modulation and protection of astrocytes could in some cases become a more effective therapeutic approach than the attempts to directly modify neuronal function or to directly protect neurons from various insults or degeneration.
